# The edible plant microbiome: evidence for the occurrence of fruit and vegetable bacteria in the human gut

**DOI:** 10.1080/19490976.2023.2258565

**Published:** 2023-09-23

**Authors:** Wisnu Adi Wicaksono, Tomislav Cernava, Birgit Wassermann, Ahmed Abdelfattah, Maria J. Soto-Giron, Gerardo V. Toledo, Suvi M. Virtanen, Mikael Knip, Heikki Hyöty, Gabriele Berg

**Affiliations:** a Institute of Environmental Biotechnology, Graz University of Technology, Graz, Austria; b School of Biological Sciences, Faculty of Environmental and Life Sciences, University of Southampton, Southampton, UK; c Leibniz Institute for Agricultural Engineering and Bioeconomy (ATB), Potsdam, Germany; d Solarea Bio, Cambridge, MA, USA; e Finnish Institute for Health and Welfare, Helsinki, Finland; f Center for Child Health Research, Tampere University and Tampere University Hospital, Tampere, Finland; g Faculty of Social Sciences, Tampere University, Tampere, Finland; h Research, Development and Innovation Center, Tampere University Hospital, Tampere, Finland; i Research Program for Clinical and Molecular Metabolism, Faculty of Medicine, University of Helsinki, Helsinki, Finland; j Pediatric Research Center, Children’s Hospital, University of Helsinki, Helsinki, Finland; k Faculty of Medicine and Health Technology, Tampere University, and Fimlab Laboratories, Tampere, Finland; l Institute for Biochemistry and Biology, University of Potsdam, Potsdam, Germany

**Keywords:** Plant microbiome, fruit, and vegetable, metagenome-assembled genomes, gut microbiome

## Abstract

Diversity of the gut microbiota is crucial for human health. However, whether fruit and vegetable associated bacteria contribute to overall gut bacterial diversity is still unknown. We reconstructed metagenome-assembled genomes from 156 fruit and vegetable metagenomes to investigate the prevalence of associated bacteria in 2,426 publicly available gut metagenomes. The microbiomes of fresh fruits and vegetables and the human gut are represented by members in common such as *Enterobacterales, Burkholderiales*, and *Lactobacillales*. Exposure to bacteria via fruit and vegetable consumption potentially has a beneficial impact on the functional diversity of gut microbiota particularly due to the presence of putative health-promoting genes for the production of vitamin and short-chain fatty acids. In the human gut, they were consistently present, although at a low abundance, approx. 2.2%. Host age, vegetable consumption frequency, and the diversity of plants consumed were drivers favoring a higher proportion. Overall, these results provide one of the primary links between the human microbiome and the environmental microbiome. This study revealed evidence that fruit and vegetable-derived microbes could be found in the human gut and contribute to gut microbiome diversity.

## Introduction

The composition and function of the human gut microbiota are closely linked to our health during our whole lifespan. Gut microbiota assembly is a complex process involving microbial seeding and succession driven by ecological forces and subject to environmental conditions.^1^ Microbiome assembly starts during birth, and babies acquire their first microbiota inoculum from their mother and other humans as well as the local environment.^2,3^ The development of the gut microbiota during early life depends on breast-feeding and environmental exposures and is critical for immune system development and long-term health outcomes.^4–6^ Related to this, a growing body of evidence has shown that changes in the composition and diversity of the gut microbiota are linked to metabolic disorders such as obesity and type 2 diabetes mellitus,^4,7^ and chronic diseases such as asthma and type 1 diabetes.^6,8^ Hence, it is of utmost importance to understand the factors influencing the composition and diversity of the gut microbiota.

Diet is a major driving factor shaping the composition of the gut microbiota. The impact of food choices, food and nutrient intake, different diet styles, and nutrition on the gut microbiome have been studied.^9,10^ During early life, the cessation of breastfeeding and the introduction of solid foods impact the maturation of the gut microbiome.^5,11,12^ Among solid foods, fruit, and vegetables are commonly consumed during early childhood. Fruits and vegetables do not only contain nutrients and bioactive plant secondary metabolites that are known to influence the gut microbial diversity and composition,^13^ but also a high diversity of microorganisms.^14,15^ Therefore, it was hypothesized that fruits and vegetables serve as the main direct sources of environmental microbiota^14,16^ and may modulate the composition and functionality of our gut microbiomes.^17^ However, there has been no study to date to investigate the potential transfer of the natural fruit and vegetable microbiota to the human gut. Moreover, fruit and vegetable associated bacteria are likely underrepresented in the human gut because large-scale food microbiome metagenomes are needed.

**Figure 1.**
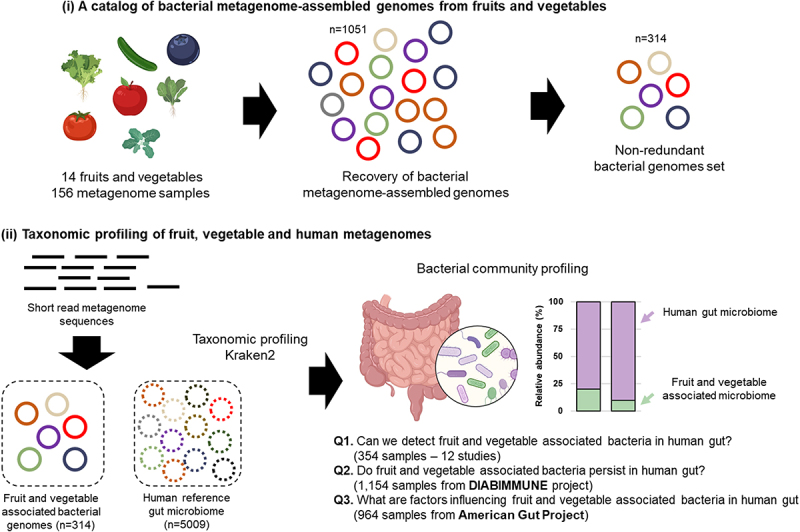
Overview of the bioinformatic workflow for the recovery of metagenome-assembled genomes (MAGs) from fruits and vegetables and the detection of their presence in the gut. Using a total of 2,426 human gut metagenomes, this study attempted to address three research questions Q1 - can we detect fruit and vegetable associated bacteria in the human gut? Q2 - do fruit and vegetable associated bacteria persist in the human gut? Q3 - what are factors influencing fruit and vegetable associated bacteria in the human gut?

The general questions to be answered are (i) Do fruit and vegetable associated bacteria contribute to the overall diversity of the gut microbiome? (ii) do these organisms persist over time in the human gut? and (iii) do specific factors including host age, frequency of fruit and vegetable consumption, and the diversity of consumed fruits and vegetables influence the fruit- and vegetable associated microbial communities in the human gut microbiome? To address these questions, we used a combination of bioinformatics tools to reconstruct representative genomes of fruit- and vegetable-associated bacteria from 156 fruit and vegetable metagenomes (Figure 1). The reconstructed genomes were used as a basis to investigate the prevalence of fruit- and vegetable-associated bacteria in 2,426 publicly available gut metagenomes including a large-scale longitudinal dataset. This approach provides an unprecedented opportunity to characterize the importance of indigenous fruit and vegetable microbiota, a neglected potential reservoir of environmental microbiota for the human gut. To the best of our knowledge, this is the first study to provide a foundation for the potential role of fruit- and vegetable-associated bacteria and fresh vegetable and fruit consumption on the development of the human gut microbiota.

## Results and discussion

### Fruit and vegetable associated bacteria are detectable in the human gut

**Figure 2.**
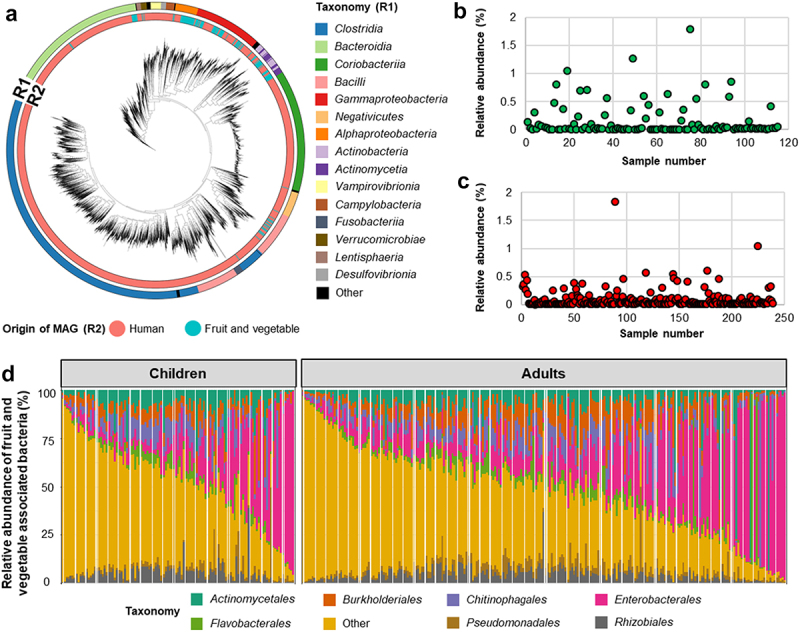
Taxonomical classification of plant and human-associated bacteria and their abundance in the human gut. Phylogenetic tree showing the taxonomical classification of fruit and vegetable and human gut-associated bacteria (a). Different colors in ring 1 (R1) indicate bacterial taxonomy and ring 2 (R2) indicates the origin of the MAGs. Relative abundance of fruit and vegetable associated bacteria and their abundance in children (b) and adults (c). Bar chart showing the relative abundance of fruit and vegetable associated bacterial composition at order level in the human gut (d). Relative abundance of fruit and vegetable associated bacteria was obtained by using datasets that contained reads that only mapped to fruit and vegetable associated bacterial genomes. Different colors represent different order-level classifications. Less abundant bacterial orders were included in “others”.

To define microbial genomes associated with fruits and vegetables, we performed metagenomic assembly and binning on 156 fruit and vegetable metagenomes, and recovered a total of 1,023 MAGs (metagenome-assembled genomes). Of the 1,023 MAGs, a total of 314 non-redundant MAGs with completeness higher than 70% and less than 10% contamination were obtained (Supplementary Table S1). In contrast to the recent human reference gut microbiome catalog, where more than half of the MAGs were assigned to the bacterial classes *Clostridia* (*n* = 2056), *Bacteroidia* (*n* = 750), and *Coriobacteria* (*n* = 716), the obtained non-redundant MAGs recovered from fruits and vegetables primarily belonged to *Gammaproteobacteria* (*n* = 98), *Alphaproteobacteria* (*n* = 78), and *Actinomycetia* (*n* = 76, Figure 2a). An overview of the taxonomic profile at genus level revealed that 31 genera, including *Pseudomonas*, *Sphingomonas*, *Aeromicrobium*, and *Pantoea* had representatives in both the human gut and the fruit and vegetable microbiomes, whereas genomes from 79 genera, including *Microbacterium*, *Nocardioides*, and *Brevundimonas*, could only be constructed from fruit and vegetable samples and not from human gut metagenome samples.

Fruit and vegetable associated bacteria harbor potentially beneficial functions for human health. Targeted analyses indicated the presence of genes known to positively affect human health, including the production of vitamin B, vitamin K, and short-chain fatty acids (Supplementary Data S1). A total of 128 MAGs harbored the *cobC* gene that encodes a cobalamin (vitamin B12) biosynthetic protein. This gene was highly prevalent in MAGs that belong to the bacterial orders *Sphingomonadales* (44.8%), *Rhizobiales* (46.4%), and *Pseudomonadales* (34.6%). Moreover, genes involved in vitamin K2 biosynthesis, i.e., *menABCDEF*, were also detected in MAGs assigned to the bacterial orders *Actinomycetales*, *Pseudomonadales*, and *Propionibacteriales*. Genes involved in short-fatty acid metabolism, such as acetate, butyrate, and propionate, were also present in the MAGs. For instance, a gene encoding acetate kinase (*ackA*) was detected in 71 MAGs mostly affilitated to *Actinomycetales* or *Enterobacterales*. Genes encoding propionate CoA-transferase and butyrate kinase that are involved in propionate and butyrate biosynthesis, respectively, were only detected in a small number of MAGs (*n* = 12 and *n* = 1, respectively) that belong to *Burkholderiales* and *Rhizobiales*.

We next used these 314 non-redundant MAGs as a reference to detect fruit and vegetable-derived microbes in the human gut microbiome We found that fruit- and vegetable-associated bacteria were consistently present at a low abundance in the human gut. First, we analyzed 354 human gut metagenome datasets from 12 studies to evaluate the presence of fruit and vegetable associated bacteria in the human gut. Using Kraken2 taxonomic profiling, on average, 88.9% of total metagenomic reads were mapped to the combined human and fruit and vegetable bacterial genome catalogs. The abundance of fruit- and vegetable-associated bacteria ranged between 0.004% and 3.604% in the gut microbiome of children and 0.003–3.122% in the gut microbiome of adults (Figure 2b, c). In alignment with a previous study, David and colleagues^9^ suggested that foodborne microbes survive in the digestive system and could be metabolically active after consuming plant matter and dairy products. After subtracting only fruit and vegetable microbial reads, compositionally, fruit and vegetables associated bacteria found in the human gut were dominated by the bacterial orders *Enterobacterales* (mean of relative abundance 20.1% in children and mean of relative abundance 22.5% in adults), *Burkholderiales* (mean of relative abundance 7.4% in children and mean of relative abundance 9.1% in adults), *Actinomycetales* (mean of relative abundance 7.8% in children and mean of relative abundance 7.6% in adults) and *Lactobacillales* (mean of relative abundance 11.9% in children and mean of relative abundance 8.5% in adults) which were represented by the bacterial genera *Erwinia*, *Lactococcus*, *Enterobacter*, and *Microbacterium* (Figure 2d). These plant-associated bacteria have been previously isolated from the human gut and linked to human health due to their roles as probiotics and pathogens.^18–20^ However, they are known for their plant beneficial effects as well as plant pathogens.^21,22^ Here, we provided evidence that fresh produce-derived bacteria can be detected in the human gut and are a component of the human gut microbiota.

Microbes occurring in soil, plants, and humans are interconnected.^14,23^ As the soil microbiome is an important reservoir of the plant microbiome, we suggest that agricultural practices not only have an effect on soil microbial diversity but also on the microbial diversity of fruits and vegetables and consequently on gut microbial diversity.^14,15^ Therefore, it will be crucial to investigate how various plant cultivation methods affect the fruit and vegetable associated microbiota. For example, previous studies revealed that naturally occurring ecosystems support more complex microbial communities than intensively managed agricultural soils.^15,24,25^ This might be reflected in the microbial diversity of fruits and vegetables. Moreover, fruits and vegetables that are cultivated hydroponically and aeroponically are expected to increase dramatically because such practices may extend the growing season and lessen exposure to soil diseases.^26,27^ However, these cultivation methods also potentially decrease bacterial diversity in fruits and vegetables due to poor microbial complexities in the production environment in comparison to natural soil systems.^28,29^ An interesting perspective would be to improve microbial diversity in the media used to grow plants in order to not only fulfill beneficial functions for the plant but also for humans consuming them.

### Fruit and vegetable associated bacteria contribute to the overall gut microbiota diversity and persist over time

**Figure 3.**
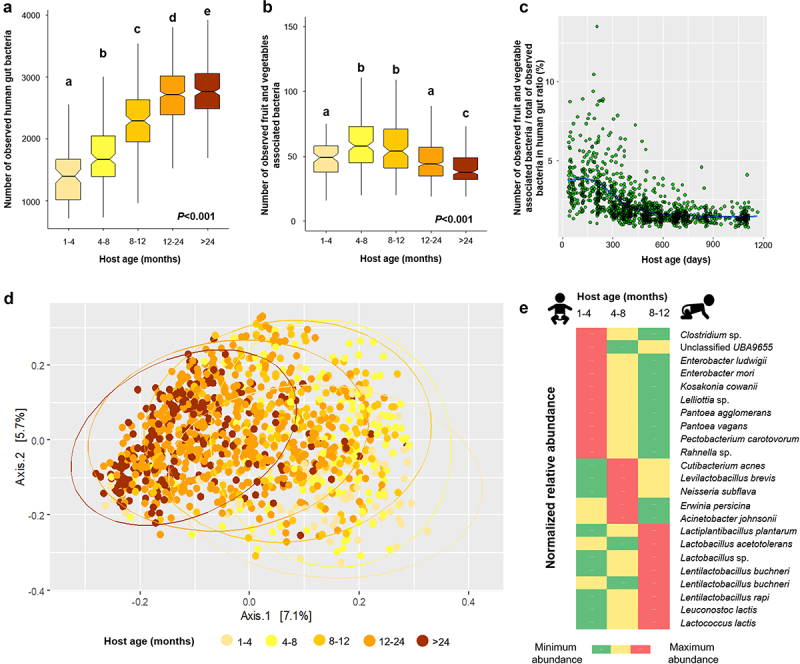
Contribution of fruit and vegetable associated bacteria to the diversity of the human gut microbiome. The box plots include the observed human gut-associated bacteria (a) and fruit and vegetable associated bacteria (b) based on the observed MAGs in the gut. The Kruskal-Wallis test followed by pairwise comparison at *P* < 0.05) within different host ages are indicated by different letters above the boxplot. The scatter plot shows the ratio of the observed fruit and vegetable associated and human gut-associated bacterial metagenome-assembled genomes (MAGs) along the age gradient (c). Community clustering of fruit and vegetable associated bacteria in the human gut is shown using a two-dimensional PCoA plot and based on a Bray–Curtis dissimilarity matrix (d). LEfSe analysis indicated fruit and vegetable associated bacterial MAGs that are enriched in different age groups (e).

Using a longitudinal study of the gut microbiome of children based on shotgun metagenome sequencing of monthly stool samples (DIABIMMUNE cohort), we further attempted to identify an overall contribution of fruit- and vegetable-associated bacteria to the overall bacterial diversity in the human gut. We observed that the bacterial diversity in the human gut significantly increased along with host age (Kruskal-Wallis test *- P* < .001, Figure 3a). Interestingly, the diversity of fruit and vegetable associated bacteria increased from 1-month-old to 12-month-old subjects, but then decreased from this point onward reaching the lowest levels in subjects older than 24 months (Kruskal-Wallis test *- P* < .001, Figure 3b). Besides breastfeeding, the introduction of solid food, i.e., fruits and vegetables, is an early life event that contributes to changes and development of the gut microbiome.^30,31^ In the DIABIMMUNE cohort, some of the children were already introduced to root vegetables (*n* = 71 of 269), fruit (*n* = 81 of 269), and vegetables (*n* = 25 of 269) during the first 4 months (Supplementary Table S2). Moreover, common cooking practices to prepare homemade baby foods might not completely eliminate the indigenous plant microbiota.^32^ Because foodborne microbes can survive in the digestive tract,^9,33^ we speculate that an increase in the number of detected plant-associated bacteria in gut metagenomes of children occurs due to the transfer and colonization of the indigenous plant microbiota from home-made or raw fruit or vegetables that were consumed during the early weaning period. Fruit and vegetable associated bacteria contributed to an average of 2.2% (min: 0.8% and max: 13.6%, Figure 3c) of the overall bacterial diversity in the human gut.

Fruit and vegetable associated bacterial communities in the human gut changed over time along with the host age, visible at a clear clustering according to the gradient of age in the PCoA plot (Figure 3d). PERMANOVA assessments indicated that host age affected the fruit and vegetable associated bacterial community structure (*P* = .001) but only explained 5.3% of the bacterial variation (Figure 3d). Interestingly, countries of origin of the infants also affected the fruit and vegetable associated bacterial community structure to a lesser degree (*P* = .001, R^2^ = 2.2%). This result could be due to different complementary feeding patterns between infants from different countries.^34^ Bacterial taxa that enriched along the gradient were mostly Gram-positive bacteria, especially the bacterial genera *Lentilactobacillus*, *Lactobacillus*, and *Lactococcus*, which replaced the early life-dominating bacterial genera *Pantoea*, *Erwinia*, and *Acinetobacter* (Figure 2d, Supplementary Figure S1A). *Pantoea agglomerans* strains originating from plants are equally able to colonize human hosts when compared to clinical strains.^35,36^ We hypothesize that due to the presence of oxygen in the newborn gut,^37^ facultative anaerobic *Gammaproteobacteria* i.e., *Pantoea*, *Erwinia*, and *Acinetobacter* which originate from fruit and vegetables can colonize in the gut of infants during the early weaning period. Then, due to the reduced oxygen concentration,^38^ anaerobic bacteria, i.e., *Lentilactobacillus* and *Lactobacillus*, emerge during the 8^th^ to 12^th^ month.

During infancy, the ecological succession of the gut microbiota is a dynamic process and then reaches a stable phase during childhood.^5,38^ This pattern was confirmed in the present study, where the human-associated bacterial composition became more homogenous along with the increase in host age (Supplementary Figure S1B). The fruit and vegetable associated bacterial composition in the human gut became more heterogeneous (i.e., higher divergence with respect to the median profile) along with the increase in host age (Supplementary Figure S1C). This might be explained by a more diverse food variety that is normally given with increasing infant age,^39^ which can also vary between individuals, leading to the observed heterogeneity associated with host age.

### Vegetable consumption frequency and the diversity of plants that are consumed affect fruit and vegetable associated bacterial richness in the human gut

We hypothesized that the frequency and diversity of fruits and vegetables that are consumed might influence fruit- and vegetable-associated bacteria richness in the human gut. To test this hypothesis, we used a dataset from the American Gut Project, a large citizen science open platform study that collected self-reported dietary data (FFQs) and fecal samples.^40^ A total of 746 samples had more than 500,000 reads that were assigned as bacterial using Kraken2. These samples were kept for further analysis to examine associations between gut bacterial diversity and plant consumption frequency and diversity.

**Figure 4.**
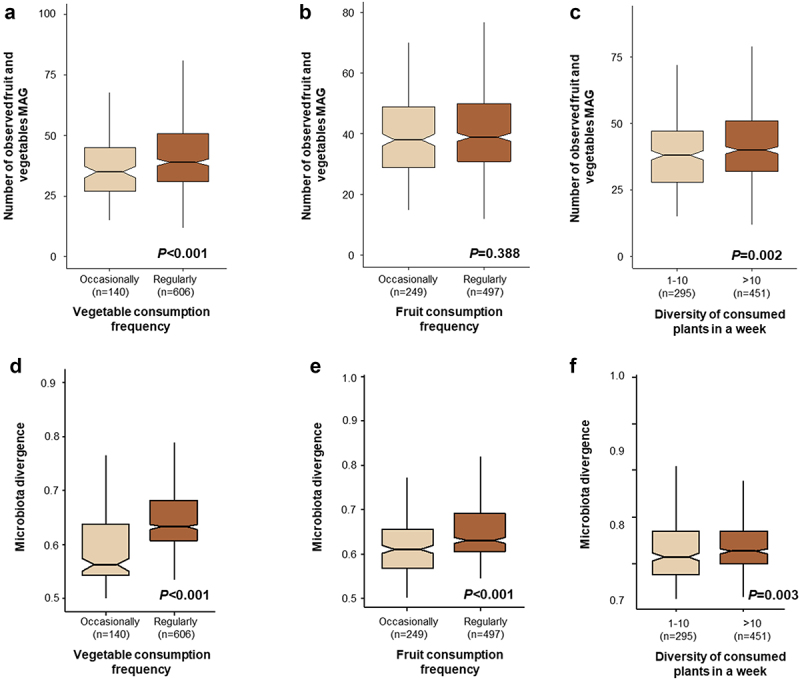
Impact of vegetable consumption frequency, fruit consumption frequency, and diversity of consumed plants in a week on the number of observed fruit and vegetable associated bacteria and overall heterogeneity in fruit and vegetable bacterial community composition. The box plots show the observed fruit and vegetable associated MAGs in the gut (a-c). The additional plots show the overall heterogenicity in fruit and vegetable associated MAG composition in the gut (d-f).

Our analysis revealed that richness of fruit and vegetable associated bacteria in the human gut is associated with frequency and diversity of vegetables consumed by the subjects. For alpha diversity analysis, we calculated the number of detected fruit and vegetable associated MAGs in the human gut after subsampling the datasets to 500,000 reads. The number of detected fruit and vegetable associated MAGs was higher in the subjects that regularly consumed vegetables and those that eat more than 10 plants weekly in comparison to other groups (Kruskal-Wallis test *- P* < .05, Figures 4a & 4c). However, fruit consumption frequency did not have a significant effect on the number of detected fruit and vegetable associated MAGs (Kruskal-Wallis test *- P* = .388, Figure 4b). We repeated the alpha diversity analysis by subsampling the dataset from 300,000 to 5,000,000 reads and found a consistent result (Supplementary Figure S2). Moreover, increased vegetable consumption frequency and diversity of consumed plants also increased relative abundance of fruit and vegetable associated bacteria in the human gut (Supplementary Figure S3). Here, we suggest that an increase in bacterial richness and relative abundance is potentially due to increased consumption of vegetables and more diverse vegetables. Aligned to our findings, rural Bedouins that regularly consume vegetables, fruits, and homemade fermented dairy products had a significantly higher bacterial diversity in comparison to urban Saudis, who reported eating vegetables and fruit just 1–2 times per week.^41^ A similar notion was also previously shown by Milani and colleagues^33^ who demonstrated colonization of dairy cattle-associated bacteria in the human gut due to consumption of Parmesan cheese daily.

Comparing the divergence across the groups that had different consumption habits of fruits and vegetables and consumed a different diversity of plants revealed that the group that more frequently consumed fruits and vegetables as well as the group that consumed more diverse plants exhibited higher divergence values (Figure 4c–f). Fruits and vegetables are known to naturally contain a diverse microbiome, often with high cell numbers, that includes beneficial, pathogenic, and spoilage microbes.^14^ Hence, consuming a wider variety of vegetables likely leads to exposure to a wider variety of microorganisms. Recently, exposure to indoor plant leaves has been confirmed to lead to the transmission of plant-associated bacteria to the human skin.^42^ Exposure to diverse bacteria via fruit and vegetable consumption could have a significant impact on the genetic diversity of the gut microbiota, in particular during childhood. Interestingly, Soto-Giron et al.^16^ showed high genetic diversity and specific functions of plant-associated strains of *Pseudomonas* and *Lactobacillus*, and specific functions associated with probiotic-linked mechanisms. Limited exposure to diverse microbiota may influence immune system development. Indeed, well-designed intervention trials and large cohort studies focused on investigating the impact of consuming fresh produce and its associated bacteria during early life on the gut microbiota development are needed to produce robust insights into the potential function and contribution of ingested food microorganisms.

## Conclusions

In conclusion, this study provides the first evidence that fruit and vegetables represent a source of the developing gut microbiota. Intake of plant-associated bacteria through fruit and vegetable consumption is one of the main connections between the human microbiome and the environmental microbiome. Therefore, any factors that influence the indigenous fruit and vegetable microbiota, i.e., farming practices, breeding, and post-harvest treatments may directly/indirectly affect the gut microbiota composition. This is important because human activities have been already linked to a shift of diversity and evenness of the plant microbiota, which is also characterized by a decrease of host specificity and an increase of r-strategic microbes (fast-growing generalists and better adapted to environmental changes), pathogens, hypermutators, and antimicrobial resistance (AMR).^43^ In contrast to r-strategists, K-strategists have slower development rates and are better at utilizing particular ecological niches. The latter provide a rich source of novel functions and are crucial for maintaining ecological stability. As suggested previously, due to human activities, r-strategic microbes will become more prevalent while K-strategists will continue to gradually disappear.^43^ Examples reflecting the impact of global agriculture on fruit microbiomes were mentioned by Wassermann et al.,^44^ identifying a storage-specific resistome in exported apples, and by Wicaksono et al.,^45^ showing the impact of cultivation on the apple and blueberry microbiota and enrichment of r-strategic microbes. On the other hand, our study provides the basis to develop joint solutions for plant and human health by application of probiotics as recently proposed.^46^ Plant-derived probiotics can be applied in the field, post-harvest,^47^ or even extracted from fruits and vegetables.^16^ Overall, this study provides the first evidence of the interconnection between the plant and the gut microbiome. The connection is established mainly by members of *Enterobacterales, Burkholderiales*, and *Lactobacillales*, which, interestingly, harbor members of both plant and human probiotics. The results also highlight the importance to maintain plant-associated microbiota diversity in order to preserve it as a natural source for the development of human gut microbiota.^46^

## Methods

### Study design

To evaluate the presence and persistence of fruit- and vegetable-associated bacteria in the human gut, and the factors that might influence these communities, we first generated a bacterial genome collection from six previously published and two in-house fruit and vegetable metagenomic datasets (described below); in total, 156 samples were obtained. We focused only on metagenomes from the edible parts of fruits and vegetables. This genome collection served as a reference database to map gut metagenome reads and estimate the relative abundance of the corresponding fruit and vegetable associated bacteria in the human gut. The study design is outlined in Figure 1. First, the presence of fruit- and vegetable-associated bacterial genomes in the human gut was assessed based on 354 shotgun metagenome datasets from 12 studies across different cohorts. Second, to investigate the persistence of plant-associated bacteria in the human gut over time, we focused on the DIABIMMUNE project (https://diabimmune.broadinstitute.org), a longitudinal study representing the development of the microbiome in infants. Third, to analyze whether the consumption frequency and the diversity of vegetables consumed influence plant-associated bacterial community structures and diversity in the human gut, we utilized a shotgun metagenome dataset from the American Gut project, a large citizen science open platform study that collected self-reported dietary data (FFQs) and fecal samples.^40^

### Metagenome assembly and reconstruction of bacterial metagenome-assembled genomes from fruits and vegetables

In total, 156 samples were obtained, spanning six public datasets (PRJEB33440, PRJNA476799, PRJNA291749, PRJNA506850, PRJNA593573, and PRJNA734564, Supplementary Table S3) and two in-house datasets. These samples originated from different fruits and vegetables, including radish (*Raphanus raphanistrum* subsp. *sativus*), lettuce (*Lactuca sativa*), apple (*Malus domestica*), spinach (*Spinacia oleracea*), blueberry (*Vaccinium corymbosum*), bayam (*Amaranthus* spp.), choy sum (*Brassica rapa* var. *parachinensis*), kai lan (*Brassica oleracea* var. *Alboglabra*), cucumber (*Cucumis sativus*), melon (*Cucumis melo* var. Cantalupo), kelp (*Laminaria abyssalis*), and others (lotus - *Lotus aduncus* , tomato - *Solanum lycopersicum*, olive - *Olea europaea*, black mission figs – *Ficus carica*).

To increase the recovery of bacterial metagenome-assembled genomes (MAGs) from the fruit and vegetable metagenomes, two binning methods, i.e., Maxbin2 v2.2.7 and MetaBAT2 v2.12.1^48,49^ were used. Multiple bins recovered with these binning methods were aggregated using DASTool v1.1.1^50^ with the parameter: –score_threshold 0.3. The metagenome-assembled genomes were then dereplicated using dRep v2.2.3^51^ to obtain a non-redundant fruit and vegetable metagenome-assembled bacterial genome dataset. Finally, the quality of MAGs was assessed using CheckM v1.0.13.^52^ Only medium-quality MAGs according to the current with at least 70% completeness and less than 10% contamination were kept for further analyses. Taxonomical information of each MAG was obtained using GTDB-Tk v1.4.1.^53^ A phylogenetic tree was constructed using PhyloPhlAn v3.0.^54^ Subsequently, the phylogenetic tree was visualized using the interactive tree of life software (iTOL).^55^ We used DRAM^56^ to predict protein-coding sequences and perform gene annotation of fruit and vegetable associated bacterial genomes

### Implementation of publicly available human metagenomes

First, we selected 354 human gut metagenome datasets from 12 gut microbiome studies (Supplementary Table S4) to detect the presence of fruit- and vegetable-associated bacteria in the human gut across different cohorts. We analyzed stool metagenomes from “Children” (1 month to 12 years) and “Adults”. To investigate the persistence of plant-associated bacteria in the human gut over time, we further retrieved a longitudinal study representing the development of the microbiome in infants from the DIABIMMUNE project (https://diabimmune.broadinstitute.org). For three years, DIABIMMUNE collected stool samples, occasional blood samples, and regular questionnaires on the early life experiences of Finnish, Estonian, and Russian children. The dataset that consists of stool metagenomes (*n* = 1,154) from 269 subjects was published previously.^8,57^ The samples were stratified by infant age group (group 1 to group 5) defined as follows: group 1: infants with age between 1 and 4 months, group 2: infants with age between 4 and 8 months, group 3: infants with age between 8 and 12 months, group 4: infants with age between 12 and 24 months, and group 5: infants with age above 24 months. The American Gut project (*n* = 964 metagenome)^40^ was utilized to correlate microbiome community data to the consumption of fruit and vegetables and the diversity of vegetables that are consumed by the cohorts.

### Taxonomic profiling of fruit, vegetable, and human metagenomes

Taxonomic profiling was applied to the abovementioned 156 fruit and vegetable metagenomes as well as 2,426 human gut metagenomes. The resulting data was then subjected to taxonomic profiling using Kraken2 v2.0.9 to classify individual metagenomic reads by mapping all *k*-mers to the lowest common ancestor (LCA) of all reference genomes.^58^ We used a non-redundant fruit and vegetable metagenome-assembled bacterial genome dataset as described above as reference. This genome set was combined with the Human Reference Gut Microbiome.^59^ Therefore, two bacterial genome groups were implemented 1) those that originated from fruit and vegetables and 2) those that are indigenous to the human gut microbiota. This combined genome set was used as a database which was constructed by Struo2 v.2.2.2^60^ for taxonomic profiling within human metagenomes using Kraken2.

### Statistical analysis

Statistical analysis and creation of graphs were conducted in RStudio^61^ unless stated otherwise. The bacterial community analysis was performed using phyloseq R packages.^62^ To account for uneven sequencing depth, the datasets were normalized by rarefying to the same sequencing depth and using MetagenomeSeq’s cumulative sum scaling (CSS)^63^ for alpha and beta diversity analysis, respectively. Significant differences in alpha diversity estimates using the number of detected bacterial genomes were analyzed by a non-parametric (ranked-based) Kruskal – Wallis test. To investigate the effect of host age, fruit and vegetable diversity, and consumption frequency on the fruit and vegetable associated bacterial community structures in the human gut, we only used datasets that contained reads that mapped only to fruit and vegetable associated bacterial genomes. The normalized Bray – Curtis dissimilarity matrix was subjected to permutational ANOVA (PERMANOVA) to test for significant effects of factors on bacterial community structures. To estimate the variability in community structures within the respective groups, we used the divergence function from the “microbiome R“ package. This function calculates divergence within groups with respect to the median profile.^64^ Finally, we used a linear discriminant analysis effect size (LEfSe)^65^ to identify differentially abundant bacterial taxa between different groups.

## Supplementary Material

Supplemental Material

Supplemental Material

## Data and materials availability

All data are available in the main text or the supplementary materials.
