# Supplementary material for: The edible plant microbiome: evidence for the occurrence of fruit and vegetable bacteria in the human gut
Source: Gut Microbes. 2023 Sep 23;15(2):2258565. doi: 10.1080/19490976.2023.2258565 (PMC10519362; doi:10.1080/19490976.2023.2258565)
Supplement: Supplemental Material [file KGMI_A_2258565_SM2165.docx]

**Supplementary Materials for**

**The edible plant microbiome: Evidence for the occurrence of fruit and vegetable bacteria in the human gut**

**Wisnu Adi Wicaksono^1*#^, Tomislav Cernava^1,2^, Birgit Wassermann^1^, Ahmed Abdelfattah^1,3^, Maria J. Soto-Giron^4^, Gerardo V. Toledo^4^, Suvi M. Virtanen^5,6,7,8^, Mikael Knip^9,10^, Heikki Hyöty^11^, Gabriele Berg^1,3,12#^**

Corresponding authors: Wisnu Adi Wicaksono and Gabriele Berg

E-mail: wisnu.wicaksono@tugraz.at; gabriele.berg@tugraz.at

**The file includes:**

Supplementary Figs. S1 to S3

Supplementary Tables S1 to S4

**Other Supplementary Materials for this manuscript include the following:**

Supplementary Data S1


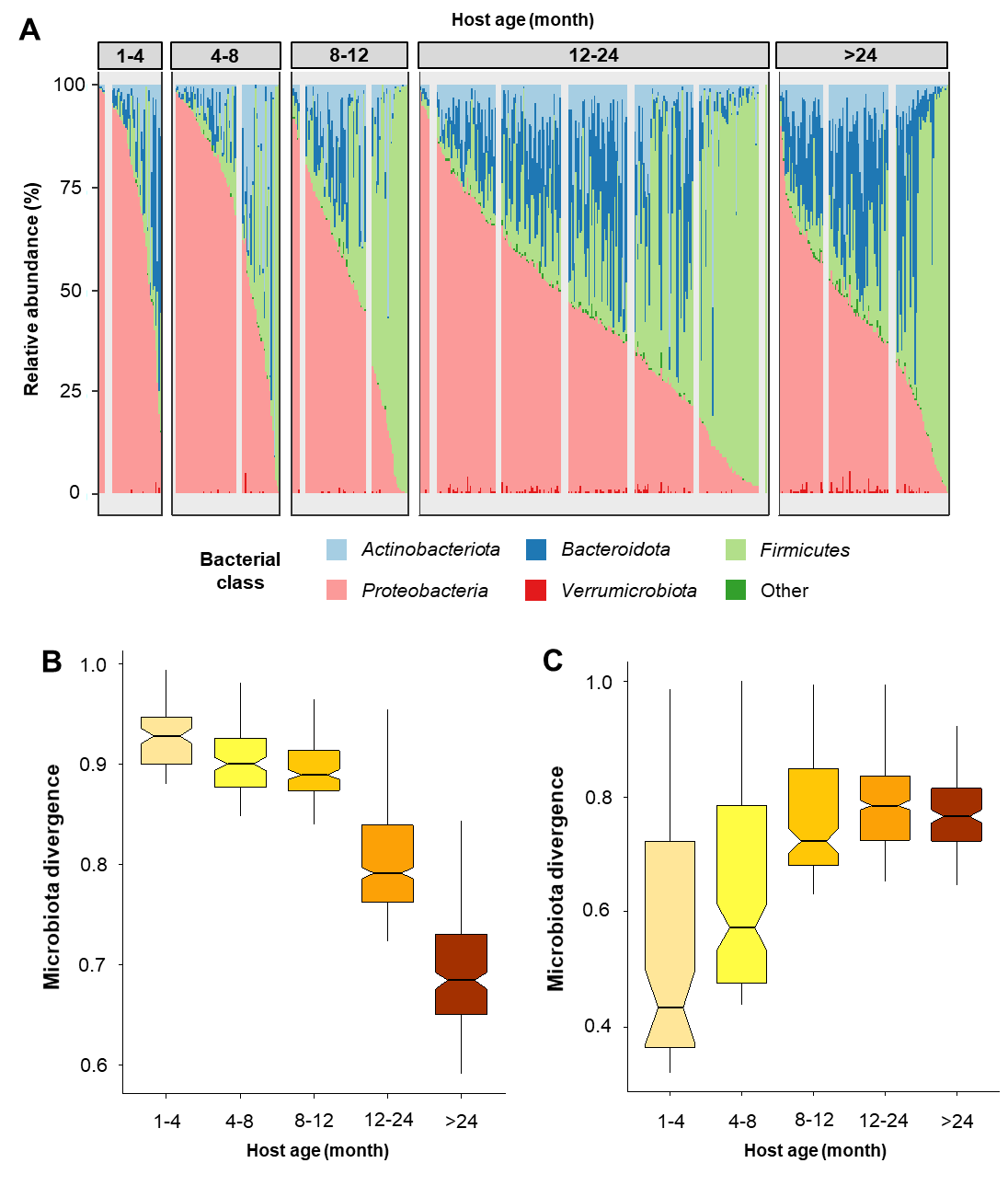


Supplementary Figure S1

**Impact of host age on overall heterogeneity in fruit and vegetable bacterial community composition.**Relative abundance of fruit and vegetable associated bacteria along the age gradient were obtained by using datasets that contain reads mapped only to fruit and vegetable associated bacterial genomes (A).

The box plots show the overall heterogenicity in human associated (B) and fruit and vegetable associated MAGs (C) composition in the gut along the age gradients.


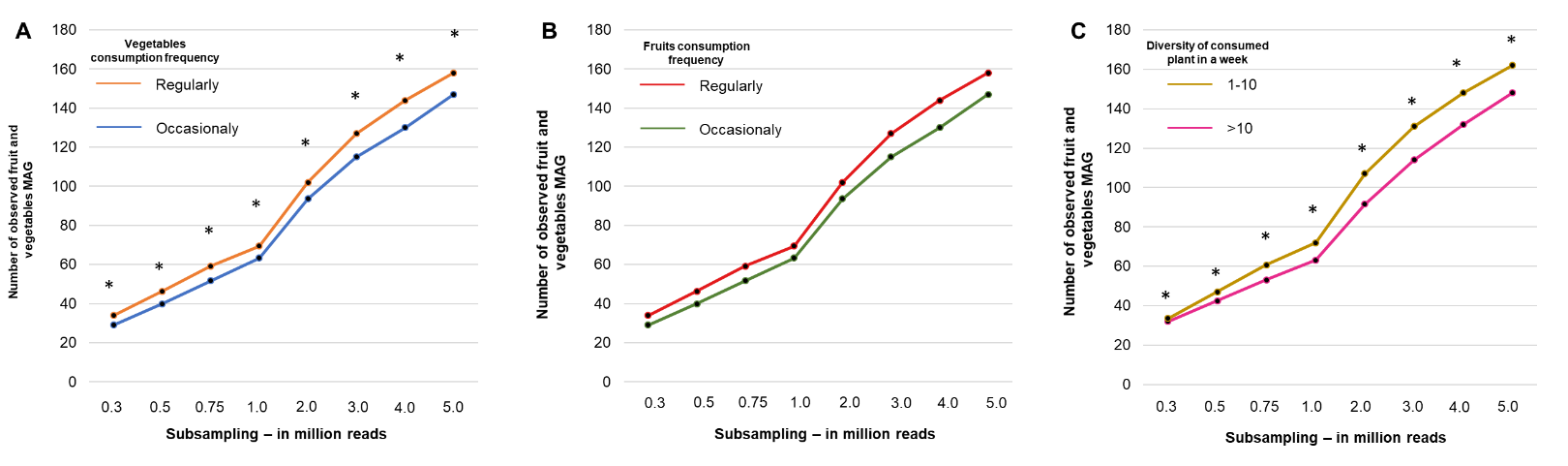


Supplementary Figure S2

**Impact of vegetable consumption frequency (A), fruit consumption frequency (B), and diversity of consumed plants in a week (C) on number of observed fruit and vegetables associated bacteria with datasets that repeatedly subsampled from 300,000 to 5,000,000 reads.** Asterix indicates a significant difference between groups (Kruskal-Wallis test *- P*<0.05).


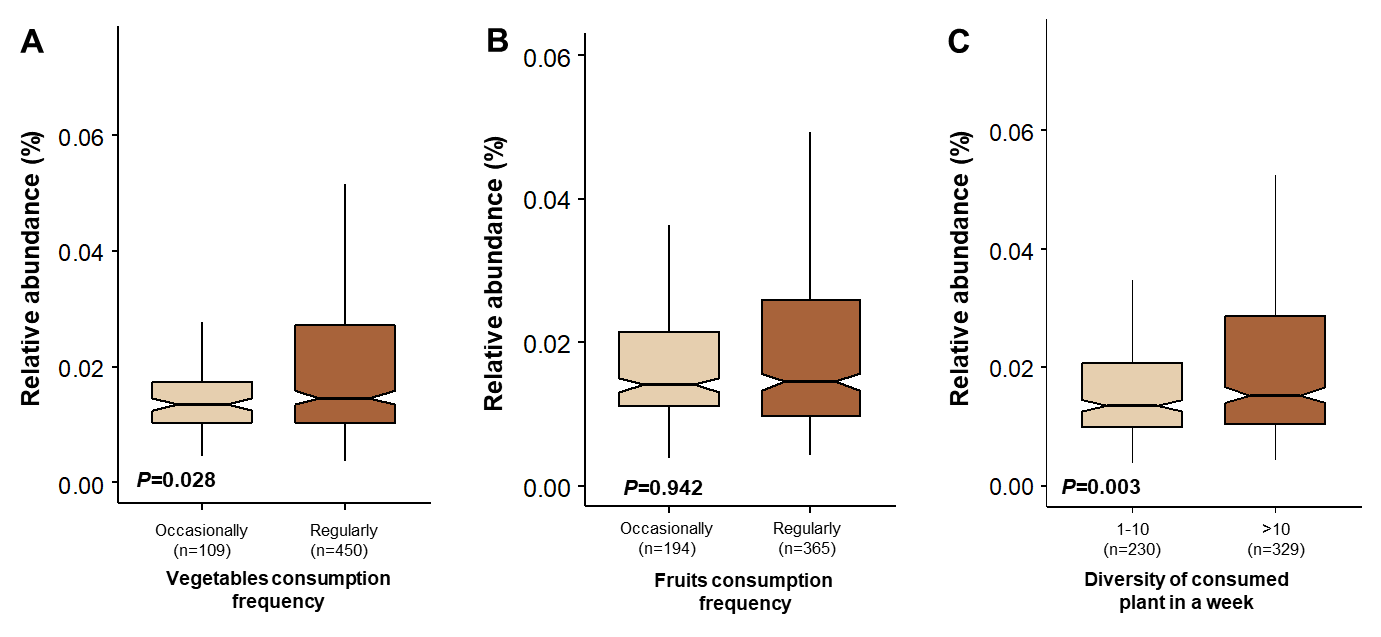


Supplementary Figure S3

**Impact of vegetable consumptions frequency (A), fruit consumptions frequency (B) and diversity of consumed plants in a week (C) on relative abundance of fruit and associated bacteria in the human gut.**

Supplementary Table S1.

**Detailed completeness and contamination values of bacterial MAGs that recovered from fruit and vegetable metagenomes**

| Genome ID | Completeness (%) | Contamination (%) | Taxonomical identification |
| --- | --- | --- | --- |
| GenomePlant1 | 76.08 | 1.9 | *Prevotella* |
| GenomePlant2 | 95.55 | 3.4 | *Prevotella* |
| GenomePlant3 | 81.62 | 2.05 | *Actinomyces* |
| GenomePlant4 | 98.03 | 0.2 | *Pantoea* |
| GenomePlant5 | 94.28 | 2.73 | *Reyranella* |
| GenomePlant6 | 78.62 | 3.45 | *Acinetobacter* |
| GenomePlant7 | 98.52 | 0.46 | *Pantoea* |
| GenomePlant8 | 92.33 | 0.9 | *Pseudomonas* |
| GenomePlant9 | 99.58 | 1.51 | Unc. *Xanthobacteraceae* |
| GenomePlant10 | 93.02 | 0 | *Rahnella* |
| GenomePlant11 | 86.49 | 3.23 | *Flavobacterium* |
| GenomePlant12 | 77.5 | 2.44 | *Microbacterium* |
| GenomePlant13 | 98.85 | 0.89 | *Microbacterium* |
| GenomePlant14 | 88.52 | 5.3 | *Bosea* |
| GenomePlant15 | 96.01 | 3.54 | *Afipia* |
| GenomePlant16 | 99.49 | 1.91 | *Sphingomonas* |
| GenomePlant17 | 97.83 | 0.87 | *Ochrobactrum* |
| GenomePlant18 | 97.16 | 0.82 | *Stenotrophomonas* |
| GenomePlant19 | 88.27 | 3.87 | *Brevundimonas* |
| GenomePlant20 | 97.08 | 2.29 | *Hylemonella* |
| GenomePlant21 | 97.88 | 1.26 | *Sphingomonas* |
| GenomePlant22 | 97.97 | 1.74 | *Reyranella* |
| GenomePlant23 | 98.35 | 1.82 | *Mesorhizobium* |
| GenomePlant24 | 89.37 | 1.15 | *CADEGZ01* |
| GenomePlant25 | 99.66 | 0.54 | *Sphingomonas* |
| GenomePlant26 | 75.02 | 1.45 | *Hyphomicrobium* |
| GenomePlant27 | 96.41 | 0.12 | *Levilactobacillus* |
| GenomePlant28 | 82.28 | 1.32 | *Kurthia* |
| GenomePlant29 | 75.17 | 0.31 | *Weissella* |
| GenomePlant30 | 97.79 | 1.93 | *Staphylococcus* |
| GenomePlant31 | 96.94 | 5.53 | *Pectobacterium* |
| GenomePlant32 | 71.45 | 0.75 | *Leuconostoc* |
| GenomePlant33 | 88.31 | 0.32 | *Lactobacillus* |
| GenomePlant34 | 90.59 | 3.25 | *Lactiplantibacillus* |
| GenomePlant35 | 97.37 | 1.75 | *Companilactobacillus* |
| GenomePlant36 | 98.31 | 0 | *Lentilactobacillus* |
| GenomePlant37 | 70.99 | 1.04 | *Vibrio* |
| GenomePlant38 | 73.28 | 2.26 | *Leuconostoc* |
| GenomePlant39 | 93.97 | 1.63 | *Lactococcus* |
| GenomePlant40 | 97.83 | 0 | *Phyllobacterium* |
| GenomePlant41 | 99.01 | 0.99 | *QFOI01* |
| GenomePlant42 | 78.13 | 1.85 | *Pseudomonas* |
| GenomePlant43 | 77.9 | 2.66 | *Methylobacterium* |
| GenomePlant44 | 73.84 | 3.13 | *Curtobacterium* |
| GenomePlant45 | 80.28 | 4.73 | *Lichenihabitans* |
| GenomePlant46 | 78.9 | 0.81 | *Pseudomonas* |
| GenomePlant47 | 99.92 | 3.58 | *Neisseria* |
| GenomePlant48 | 74.92 | 6.15 | *Rhodococcus* |
| GenomePlant49 | 99.09 | 1.38 | *Acinetobacter* |
| GenomePlant50 | 75.17 | 9.41 | *UBA9655* |
| GenomePlant51 | 97.54 | 0 | *Sediminibacterium* |
| GenomePlant52 | 99.67 | 0.81 | *Pantoea* |
| GenomePlant53 | 96.69 | 4.6 | *Macrococcus* |
| GenomePlant54 | 100 | 3.96 | *Exiguobacterium* |
| GenomePlant55 | 74.59 | 3.39 | *Pantoea* |
| GenomePlant56 | 81.47 | 7.47 | *Acinetobacter* |
| GenomePlant57 | 99.67 | 0.99 | *Pseudomonas* |
| GenomePlant58 | 99.44 | 0.97 | *Chryseobacterium* |
| GenomePlant59 | 99.93 | 6.41 | *Chryseobacterium* |
| GenomePlant60 | 100 | 2.21 | *Chryseobacterium* |
| GenomePlant61 | 96.48 | 1.4 | *Kosakonia* |
| GenomePlant62 | 83.55 | 6.24 | *Pedobacter* |
| GenomePlant63 | 93.25 | 3.94 | *Edaphocola* |
| GenomePlant64 | 77.38 | 2.03 | *UBA11358* |
| GenomePlant65 | 94.79 | 4.13 | *Flavobacterium* |
| GenomePlant66 | 99.67 | 1.64 | *Lelliottia* |
| GenomePlant67 | 99.53 | 4.11 | *Methylophilus* |
| GenomePlant68 | 95.8 | 7.47 | *Citricoccus* |
| GenomePlant69 | 95.18 | 3.31 | *Exiguobacterium* |
| GenomePlant70 | 96.72 | 7.7 | *Methylophilus* |
| GenomePlant71 | 80.05 | 4.82 | *Methylotenera* |
| GenomePlant72 | 100 | 0.78 | *Paenarthrobacter* |
| GenomePlant73 | 79.19 | 6.99 | *Niabella* |
| GenomePlant74 | 98.82 | 4.66 | *Pseudomonas* |
| GenomePlant75 | 94.77 | 5.06 | *Thermoactinomyces* |
| GenomePlant76 | 70.69 | 9.48 | *Methylophilus* |
| GenomePlant77 | 94.08 | 3.5 | *Caulobacter* |
| GenomePlant78 | 91.67 | 5.51 | *Mucilaginibacter* |
| GenomePlant79 | 99.97 | 2.6 | *Enterobacter* |
| GenomePlant80 | 70.94 | 9.38 | *Brevundimonas* |
| GenomePlant81 | 99.84 | 1.25 | *Caulobacter* |
| GenomePlant82 | 97.02 | 1.03 | *Pseudomonas* |
| GenomePlant83 | 94.59 | 3.28 | *Lacunisphaera* |
| GenomePlant84 | 99.73 | 1.79 | *Trinickia* |
| GenomePlant85 | 85.94 | 2.02 | *CAIMXF01* |
| GenomePlant86 | 83.42 | 2.13 | *Trinickia* |
| GenomePlant87 | 90.83 | 1.2 | *Bdellovibrio* |
| GenomePlant88 | 90.27 | 3.66 | *Caenibius* |
| GenomePlant89 | 97.56 | 0.67 | *Alkanindiges* |
| GenomePlant90 | 93.81 | 1.78 | Unc*. Burkholderiaceae* |
| GenomePlant91 | 91.38 | 1.82 | *Terracidiphilus* |
| GenomePlant92 | 96.69 | 0.51 | *Microbacterium* |
| GenomePlant93 | 79.84 | 1.12 | *Nocardioides* |
| GenomePlant94 | 99.05 | 0.91 | *Nocardioides* |
| GenomePlant95 | 79.94 | 2.29 | Unc*. Pseudonocardiaceae* |
| GenomePlant96 | 88.56 | 2.82 | Unc*. UBA8346* |
| GenomePlant97 | 81.45 | 1.35 | *Cellulomonas* |
| GenomePlant98 | 93.51 | 1.68 | *Glutamicibacter* |
| GenomePlant99 | 99.48 | 1.3 | *Sphingomonas* |
| GenomePlant100 | 84.59 | 1.29 | *Pseudomonas* |
| GenomePlant101 | 98.97 | 0.76 | *Pantoea* |
| GenomePlant102 | 72.75 | 1.64 | *Exiguobacterium* |
| GenomePlant103 | 96.25 | 0.6 | *Pseudomonas* |
| GenomePlant104 | 97.36 | 3.51 | *Sphingomonas* |
| GenomePlant105 | 78.01 | 1.32 | *Methylobacterium* |
| GenomePlant106 | 85.54 | 2.71 | *Sphingomonas* |
| GenomePlant107 | 75.45 | 0.51 | *Microbacterium* |
| GenomePlant108 | 77.73 | 2.11 | *Sphingomonas* |
| GenomePlant109 | 81.78 | 1.61 | *Mitsuaria* |
| GenomePlant110 | 94.52 | 4.06 | *Microbacterium* |
| GenomePlant111 | 87.15 | 5.69 | *Microbacterium* |
| GenomePlant112 | 85.89 | 5.44 | *Novosphingobium* |
| GenomePlant113 | 92.22 | 6.59 | *Microbacterium* |
| GenomePlant114 | 84.15 | 3.75 | *Shinella* |
| GenomePlant115 | 96.04 | 0.02 | *Edaphocola* |
| GenomePlant116 | 99.47 | 1.68 | *Lysobacter* |
| GenomePlant117 | 97.31 | 1.27 | *Agrobacterium* |
| GenomePlant118 | 98.44 | 1.83 | *Devosia* |
| GenomePlant119 | 98.65 | 0.19 | *Promicromonospora* |
| GenomePlant120 | 96.43 | 3.06 | *Microbacterium* |
| GenomePlant121 | 99.84 | 0.5 | *Brevundimonas* |
| GenomePlant122 | 81.53 | 0.49 | *Paenarthrobacter* |
| GenomePlant123 | 98.02 | 0.72 | *Pseudomonas* |
| GenomePlant124 | 94.01 | 2.02 | *Curtobacterium* |
| GenomePlant125 | 91.99 | 2.64 | *Sphingopyxis* |
| GenomePlant126 | 77.49 | 0.27 | *Pseudomonas* |
| GenomePlant127 | 92.28 | 5.78 | *Variovorax* |
| GenomePlant128 | 99.66 | 1.74 | *Lysobacter* |
| GenomePlant129 | 82.79 | 1.18 | *Caulobacter* |
| GenomePlant130 | 98.98 | 2.47 | *Sphingopyxis* |
| GenomePlant131 | 99.23 | 2.36 | *Sphingobium* |
| GenomePlant132 | 88.82 | 1 | *Edaphocola* |
| GenomePlant133 | 92.69 | 3.46 | *Neorhizobium* |
| GenomePlant134 | 97.8 | 0.8 | *Pseudomonas* |
| GenomePlant135 | 97.19 | 2.81 | *Nocardioides* |
| GenomePlant136 | 97.31 | 0.7 | *Stenotrophomonas* |
| GenomePlant137 | 98.29 | 2.06 | *Sphingomonas* |
| GenomePlant138 | 85.84 | 2.99 | *JAAZBK01* |
| GenomePlant139 | 94.76 | 4.06 | *Brevundimonas* |
| GenomePlant140 | 94.74 | 3.51 | *Curtobacterium* |
| GenomePlant141 | 98.5 | 1.87 | *Agrobacterium* |
| GenomePlant142 | 95.27 | 2.54 | *Stenotrophomonas* |
| GenomePlant143 | 97.61 | 6.65 | *Variovorax* |
| GenomePlant144 | 82.97 | 2.11 | *Xanthomonas* |
| GenomePlant145 | 83 | 0.16 | *Agrobacterium* |
| GenomePlant146 | 99.31 | 2.39 | *Novosphingobium* |
| GenomePlant147 | 94.53 | 2.79 | *Microbacterium* |
| GenomePlant148 | 99.2 | 0.68 | *Agrobacterium* |
| GenomePlant149 | 87.83 | 9.73 | *Sphingopyxis* |
| GenomePlant150 | 83.07 | 0.43 | *Phyllobacterium* |
| GenomePlant151 | 93.87 | 1.33 | *Arthrobacter* |
| GenomePlant152 | 89.08 | 2.29 | *Pelomonas* |
| GenomePlant153 | 77.39 | 0 | *Paenarthrobacter* |
| GenomePlant154 | 90.19 | 4.82 | *Aeromicrobium* |
| GenomePlant155 | 97.51 | 4.94 | *Microbacterium* |
| GenomePlant156 | 93.3 | 2.19 | *Glycomyces* |
| GenomePlant157 | 74.5 | 8.04 | *Nocardioides* |
| GenomePlant158 | 98.38 | 1.98 | *Devosia* |
| GenomePlant159 | 97.67 | 2.92 | *Lysobacter* |
| GenomePlant160 | 93.07 | 2.09 | *Brevundimonas* |
| GenomePlant161 | 75 | 1.72 | Unc*. Saccharimonadaceae* |
| GenomePlant162 | 97.32 | 2.39 | *Pedobacter* |
| GenomePlant163 | 83.63 | 1.12 | *Microbacterium* |
| GenomePlant164 | 99.18 | 2.48 | *Nocardioides* |
| GenomePlant165 | 92.18 | 1.64 | *Microbacterium* |
| GenomePlant166 | 70.59 | 4.4 | *Variovorax* |
| GenomePlant167 | 89.64 | 5.13 | *Aeromicrobium* |
| GenomePlant168 | 88.72 | 2.91 | *Arenimonas* |
| GenomePlant169 | 99.59 | 1.37 | *Sphingobium* |
| GenomePlant170 | 95.29 | 3.33 | *Methylophilus* |
| GenomePlant171 | 83.44 | 3.4 | *Aeromicrobium* |
| GenomePlant172 | 94.77 | 2.42 | *Pelomonas* |
| GenomePlant173 | 88.76 | 4.92 | *Sphingomonas* |
| GenomePlant174 | 91.85 | 6.74 | *Caulobacter* |
| GenomePlant175 | 98.82 | 0 | *Methylophilus* |
| GenomePlant176 | 84.06 | 0.25 | *Pseudomonas* |
| GenomePlant177 | 97.85 | 0.43 | *CABHOJ01* |
| GenomePlant178 | 95.08 | 6 | *Mesorhizobium* |
| GenomePlant179 | 96.1 | 5.03 | *Acidovorax* |
| GenomePlant180 | 95.67 | 0 | *Methylophilus* |
| GenomePlant181 | 86.79 | 2.84 | *Phycicoccus* |
| GenomePlant182 | 81.52 | 2.53 | *Stenotrophomonas* |
| GenomePlant183 | 97.83 | 0.24 | *Enterobacter* |
| GenomePlant184 | 95.02 | 3.85 | *Microbacterium* |
| GenomePlant185 | 89.43 | 3.77 | *Nocardioides* |
| GenomePlant186 | 98.9 | 1.02 | *Pseudomonas* |
| GenomePlant187 | 97.51 | 7.43 | *Nocardioides* |
| GenomePlant188 | 89.66 | 4.79 | *Variovorax* |
| GenomePlant189 | 98.98 | 2.78 | *Sphingopyxis* |
| GenomePlant190 | 84.74 | 0.43 | *Methylophilus* |
| GenomePlant191 | 90.07 | 3.89 | *Aeromicrobium* |
| GenomePlant192 | 82.45 | 2.24 | *Microbacterium* |
| GenomePlant193 | 99.1 | 0.29 | *Pseudomonas* |
| GenomePlant194 | 96.49 | 5.52 | *Curtobacterium* |
| GenomePlant195 | 99.27 | 1.58 | *Acidovorax* |
| GenomePlant196 | 96.17 | 0.48 | *Pedobacter* |
| GenomePlant197 | 99.11 | 2.81 | *Sphingomonas* |
| GenomePlant198 | 97.17 | 1.1 | Unc*. Sphingomonadaceae* |
| GenomePlant199 | 95.97 | 1.3 | *JAAFHU01* |
| GenomePlant200 | 91.14 | 4.9 | *Microbacterium* |
| GenomePlant201 | 96.82 | 2.13 | *Tahibacter* |
| GenomePlant202 | 98.96 | 1.7 | *Agrobacterium* |
| GenomePlant203 | 90.72 | 0.72 | *Pedobacter* |
| GenomePlant204 | 99 | 2.2 | *Acidovorax* |
| GenomePlant205 | 99.52 | 0.64 | *Pedobacter* |
| GenomePlant206 | 93.58 | 2.92 | *Asticcacaulis* |
| GenomePlant207 | 93.71 | 2.56 | *Thermomonas* |
| GenomePlant208 | 99.22 | 0.62 | *Stenotrophomonas* |
| GenomePlant209 | 98.12 | 0.72 | *Flavobacterium* |
| GenomePlant210 | 99.46 | 5.08 | *Aeromicrobium* |
| GenomePlant211 | 90.86 | 2.38 | *UBA1315* |
| GenomePlant212 | 95.46 | 0.47 | *Methylophilus* |
| GenomePlant213 | 91.8 | 8.64 | *Caulobacter* |
| GenomePlant214 | 91.84 | 1.98 | *Microbacterium* |
| GenomePlant215 | 95.26 | 1.81 | *VAYN01* |
| GenomePlant216 | 97.88 | 2.24 | *62-47* |
| GenomePlant217 | 92.02 | 4.19 | *Sphingopyxis* |
| GenomePlant218 | 95.13 | 0.53 | *Pantoea* |
| GenomePlant219 | 73.53 | 2.86 | *Glutamicibacter* |
| GenomePlant220 | 93.84 | 4.06 | *Microbacterium* |
| GenomePlant221 | 97.88 | 1.02 | *Sphingobium* |
| GenomePlant222 | 88.26 | 1.58 | *Caulobacter* |
| GenomePlant223 | 83.62 | 1.72 | *Methylophilus* |
| GenomePlant224 | 77.86 | 3.21 | *Pedobacter* |
| GenomePlant225 | 98.24 | 4.69 | *Sphingopyxis* |
| GenomePlant226 | 73.45 | 5.44 | *Aeromicrobium* |
| GenomePlant227 | 97.23 | 5.8 | *Agrobacterium* |
| GenomePlant228 | 81.84 | 3.4 | *Curtobacterium* |
| GenomePlant229 | 89.77 | 5.13 | *Aeromicrobium* |
| GenomePlant230 | 89.14 | 2.31 | *Pantoea* |
| GenomePlant231 | 98.9 | 0 | *Brochothrix* |
| GenomePlant232 | 93.37 | 0.73 | *Clostridium* |
| GenomePlant233 | 74.48 | 3.45 | *Erwinia* |
| GenomePlant234 | 98.39 | 0 | *Saccharomonospora* |
| GenomePlant235 | 98.7 | 0.74 | *Nocardioides* |
| GenomePlant236 | 98.96 | 1.49 | *Aeromicrobium* |
| GenomePlant237 | 92.45 | 2.32 | *Microbacterium* |
| GenomePlant238 | 100 | 0 | *Niabella* |
| GenomePlant239 | 82.39 | 2.93 | *Pseudoxanthomonas* |
| GenomePlant240 | 89.79 | 5.25 | *Shinella* |
| GenomePlant241 | 100 | 0.66 | *Cutibacterium* |
| GenomePlant242 | 72.71 | 0.05 | *Pseudomonas* |
| GenomePlant243 | 71.58 | 1.75 | *Pseudomonas* |
| GenomePlant244 | 96.65 | 0.12 | *Polaromonas* |
| GenomePlant245 | 77.7 | 6.06 | *Caenibius* |
| GenomePlant246 | 70.99 | 3.54 | *Microbacterium* |
| GenomePlant247 | 99.64 | 2.75 | *Sphingopyxis* |
| GenomePlant248 | 100 | 0.52 | *Brevundimonas* |
| GenomePlant249 | 89.85 | 0.12 | *Stenotrophomonas* |
| GenomePlant250 | 96.3 | 2.24 | *Devosia* |
| GenomePlant251 | 99.2 | 1.98 | *Microbacterium* |
| GenomePlant252 | 92.33 | 2.21 | *Chryseobacterium* |
| GenomePlant253 | 80.16 | 1.37 | *Saccharomonospora* |
| GenomePlant254 | 89.77 | 0.72 | *Pantoea* |
| GenomePlant255 | 86.38 | 3.83 | *CABHOJ01* |
| GenomePlant256 | 98.59 | 0.15 | *Flavobacterium* |
| GenomePlant257 | 91.47 | 0.18 | *Pseudomonas* |
| GenomePlant258 | 95.78 | 2.5 | *Acidovorax* |
| GenomePlant259 | 96.85 | 0.15 | *Pseudomonas* |
| GenomePlant260 | 85.18 | 0.5 | *Niabella* |
| GenomePlant261 | 81.93 | 1.74 | *Brevundimonas* |
| GenomePlant262 | 86.8 | 2.56 | *Sphingomonas* |
| GenomePlant263 | 99.49 | 0.51 | *Microbacterium* |
| GenomePlant264 | 73.06 | 2.44 | *Caulobacter* |
| GenomePlant265 | 81.62 | 0 | *Chryseobacterium* |
| GenomePlant266 | 98.48 | 1.43 | *Microbacterium* |
| GenomePlant267 | 97.91 | 0.09 | *Flavobacterium* |
| GenomePlant268 | 83.02 | 2.95 | *Chiayiivirga* |
| GenomePlant269 | 83.82 | 8.81 | *Nocardioides* |
| GenomePlant270 | 98.41 | 1.05 | *Sphingobium* |
| GenomePlant271 | 100 | 2.4 | *Brevundimonas* |
| GenomePlant272 | 98.42 | 0.29 | *Paenarthrobacter* |
| GenomePlant273 | 94.98 | 7.56 | *Aeromicrobium* |
| GenomePlant274 | 71.39 | 7.45 | *ALPHA2B* |
| GenomePlant275 | 99.02 | 0.49 | *Chryseobacterium* |
| GenomePlant276 | 99.13 | 1.16 | *Brevundimonas* |
| GenomePlant277 | 99.53 | 0.16 | *PHCI01* |
| GenomePlant278 | 77.86 | 3.56 | *Nocardioides* |
| GenomePlant279 | 99.22 | 2.33 | *Aeromicrobium* |
| GenomePlant280 | 99.12 | 0.05 | *Pseudomonas* |
| GenomePlant281 | 97.78 | 0.12 | *Chryseobacterium* |
| GenomePlant282 | 95.76 | 1.32 | *Paenarthrobacter* |
| GenomePlant283 | 81.21 | 0.95 | *Nocardioides* |
| GenomePlant284 | 94.01 | 1.04 | *Pseudomonas* |
| GenomePlant285 | 97.83 | 2.01 | *Lysobacter* |
| GenomePlant286 | 98.74 | 0.97 | *Brevundimonas* |
| GenomePlant287 | 93.24 | 2.49 | *Novosphingobium* |
| GenomePlant288 | 93.91 | 0.78 | *Arthrobacter* |
| GenomePlant289 | 98.15 | 2.37 | *Microbacterium* |
| GenomePlant290 | 98.37 | 2.27 | *Microbacterium* |
| GenomePlant291 | 95.97 | 0.67 | *Microbacterium* |
| GenomePlant292 | 96.65 | 0.48 | *Pedobacter* |
| GenomePlant293 | 99.43 | 2.16 | *Aeromicrobium* |
| GenomePlant294 | 95.76 | 1.11 | *Agrobacterium* |
| GenomePlant295 | 95.45 | 2.26 | *Pedobacter* |
| GenomePlant296 | 98.35 | 0.46 | *Agrobacterium* |
| GenomePlant297 | 100 | 0 | *Thermobifida* |
| GenomePlant298 | 95.6 | 1.94 | *Microbacterium* |
| GenomePlant299 | 98.46 | 2.5 | *Sphingomonas* |
| GenomePlant300 | 96.63 | 5.39 | *Aeromicrobium* |
| GenomePlant301 | 99.4 | 0.12 | *Agrobacterium* |
| GenomePlant302 | 99.28 | 1.44 | *Lysobacter* |
| GenomePlant303 | 79.3 | 1.79 | *Stenotrophomonas* |
| GenomePlant304 | 100 | 8.15 | *Rahnella* |
| GenomePlant305 | 90.79 | 3.04 | *Lactobacillus* |
| GenomePlant306 | 99.67 | 5.04 | *Janthinobacterium* |
| GenomePlant307 | 96.03 | 1.87 | *Erwinia* |
| GenomePlant308 | 95.08 | 0.33 | *Lonsdalea* |
| GenomePlant309 | 98.39 | 0.38 | *Pseudomonas* |
| GenomePlant310 | 97.01 | 1.12 | *Loigolactobacillus* |
| GenomePlant311 | 96.25 | 0 | *Lentilactobacillus* |
| GenomePlant312 | 96.56 | 0.69 | *Lentilactobacillus* |
| GenomePlant313 | 98.38 | 0.4 | *Herbaspirillum* |
| GenomePlant314 | 75.86 | 0 | *Pantoea* |

Supplementary Table S2.

**Details of age for first contact with fruits and vegetables from DIABIMMUNE cohort.**

| Age in months for first contact with fruit or berries | | No. of subject |
| --- | --- | --- |
| 1_Age_1_4 | 81 |  |
| 2_Age_4_8 | 96 |  |
| >8 | 1 |  |
| NA | 91 |  |
|  |  |  |
| Age in months for first contact with root vegetables | No. of subject |  |
| 1_Age_1_4 | 71 |  |
| 2_Age_4_8 | 103 |  |
| >8 | 2 |  |
| NA | 93 |  |
|  |  |  |
| Age in months for first contact with vegetables | No. of subject |  |
| 1_Age_1_4 | 25 |  |
| 2_Age_4_8 | 103 |  |
| >8 | 6 |  |
| NA | 93 |  |

Supplementary Table S3.

List of accession numbers of fruit and vegetable metagenome samples that used in this study

| Accesion number | Project number | Sample type |
| --- | --- | --- |
| SBP0049 | *In house* dataset | Blueberries |
| SBP0077 | *In house* dataset | Blueberries |
| SBP0199 | *In house* dataset | Blueberries |
| SBP0079 | *In house* dataset | Blueberries |
| SBP0063 | *In house* dataset | Blueberries |
| SBP0075 | *In house* dataset | Blueberries |
| SBP0076 | *In house* dataset | Blueberries |
| SBP0078 | *In house* dataset | Blueberries |
| SBP0080 | *In house* dataset | Blueberries |
| M2 | *In house* dataset | Apple |
| M3 | *In house* dataset | Apple |
| A3 | *In house* dataset | Apple |
| C3W | *In house* dataset | Apple |
| C2W | *In house* dataset | Apple |
| SBP0176 | *In house* dataset | Apple |
| A1 | *In house* dataset | Apple |
| C1W | *In house* dataset | Apple |
| M1 | *In house* dataset | Apple |
| O2S | *In house* dataset | Apple |
| O3S | *In house* dataset | Apple |
| O1S | *In house* dataset | Apple |
| A2 | *In house* dataset | Apple |
| SBP0055 | PRJNA635436 | Early Girl tomatoes |
| SBP0059 | PRJNA635436 | Baby Spinach |
| SBP0215 | PRJNA635436 | Black Mission Figs |
| ERR3427626 | PRJEB33440 | Cucumis_melo_var_cantalupo |
| ERR3427617 | PRJEB33440 | Cucumis_sativus |
| ERR3427618 | PRJEB33440 | Cucumis_sativus |
| ERR3427623 | PRJEB33440 | Cucumis_sativus |
| ERR3427620 | PRJEB33440 | Lotus_aduncus |
| ERR3427619 | PRJEB33440 | Cucumis_sativus |
| ERR3427621 | PRJEB33440 | Laminaria_abyssalis |
| SRR2177282 | PRJNA291749 | spinach |
| SRR2177285 | PRJNA291749 | spinach |
| SRR2177359 | PRJNA291749 | spinach |
| SRR2177286 | PRJNA291749 | spinach |
| SRR2177280 | PRJNA291749 | spinach |
| SRR2177283 | PRJNA291749 | spinach |
| SRR2177361 | PRJNA291749 | spinach |
| SRR2177287 | PRJNA291749 | spinach |
| SRR2177358 | PRJNA291749 | spinach |
| SRR2177251 | PRJNA291749 | spinach |
| SRR2177288 | PRJNA291749 | spinach |
| SRR2177360 | PRJNA291749 | spinach |
| SRR2177250 | PRJNA291749 | spinach |
| SRR2177284 | PRJNA291749 | spinach |
| SRR2177357 | PRJNA291749 | spinach |
| SRR2177281 | PRJNA291749 | spinach |
| SRR7414919 | PRJNA476799 | Lettuce |
| SRR7414910 | PRJNA476799 | Lettuce |
| SRR7414914 | PRJNA476799 | Lettuce |
| SRR7414917 | PRJNA476799 | Lettuce |
| SRR7414918 | PRJNA476799 | Lettuce |
| SRR7414902 | PRJNA476799 | Lettuce |
| SRR7414941 | PRJNA476799 | Lettuce |
| SRR7414911 | PRJNA476799 | Lettuce |
| SRR7414909 | PRJNA476799 | Lettuce |
| SRR7414916 | PRJNA476799 | Lettuce |
| SRR7414921 | PRJNA476799 | Lettuce |
| SRR7414927 | PRJNA476799 | Lettuce |
| SRR7414945 | PRJNA476799 | Radish |
| SRR7414950 | PRJNA476799 | Radish |
| SRR7414938 | PRJNA476799 | Lettuce |
| SRR7414922 | PRJNA476799 | Lettuce |
| SRR7414946 | PRJNA476799 | Radish |
| SRR7414906 | PRJNA476799 | Radish |
| SRR7414924 | PRJNA476799 | Lettuce |
| SRR7414925 | PRJNA476799 | Radish |
| SRR7414931 | PRJNA476799 | Radish |
| SRR7414947 | PRJNA476799 | Radish |
| SRR7414907 | PRJNA476799 | Radish |
| SRR7414913 | PRJNA476799 | Lettuce |
| SRR7414926 | PRJNA476799 | Radish |
| SRR7414932 | PRJNA476799 | Radish |
| SRR7414943 | PRJNA476799 | Radish |
| SRR7414944 | PRJNA476799 | Radish |
| SRR7414905 | PRJNA476799 | Radish |
| SRR7414939 | PRJNA476799 | Lettuce |
| SRR7414908 | PRJNA476799 | Radish |
| SRR7414912 | PRJNA476799 | Radish |
| SRR7414930 | PRJNA476799 | Radish |
| SRR7414934 | PRJNA476799 | Radish |
| SRR7414936 | PRJNA476799 | Radish |
| SRR7414937 | PRJNA476799 | Radish |
| SRR7414915 | PRJNA476799 | Lettuce |
| SRR7414933 | PRJNA476799 | Radish |
| SRR7414929 | PRJNA476799 | Lettuce |
| SRR7414935 | PRJNA476799 | Radish |
| SRR7414948 | PRJNA476799 | Radish |
| SRR7414920 | PRJNA476799 | Lettuce |
| SRR7414942 | PRJNA476799 | Radish |
| SRR7414949 | PRJNA476799 | Radish |
| SRR7414951 | PRJNA476799 | Radish |
| SRR7414903 | PRJNA476799 | Lettuce |
| SRR7414904 | PRJNA476799 | Lettuce |
| SRR7414940 | PRJNA476799 | Lettuce |
| SRR7414928 | PRJNA476799 | Lettuce |
| SRR7414923 | PRJNA476799 | Lettuce |
| SRR8448066 | PRJNA506850 | Radish |
| SRR13213290 | PRJNA506850 | Radish |
| SRR13213291 | PRJNA506850 | Radish |
| SRR13213275 | PRJNA506850 | Radish |
| SRR13213287 | PRJNA506850 | Radish |
| SRR13213284 | PRJNA506850 | Radish |
| SRR13213285 | PRJNA506850 | Radish |
| SRR13213292 | PRJNA506850 | Radish |
| SRR13213268 | PRJNA506850 | Radish |
| SRR13213270 | PRJNA506850 | Radish |
| SRR13213267 | PRJNA506850 | Radish |
| SRR13213274 | PRJNA506850 | Radish |
| SRR13213277 | PRJNA506850 | Radish |
| SRR13213278 | PRJNA506850 | Radish |
| SRR13213280 | PRJNA506850 | Radish |
| SRR13213281 | PRJNA506850 | Radish |
| SRR13213286 | PRJNA506850 | Radish |
| SRR13213289 | PRJNA506850 | Radish |
| SRR13213269 | PRJNA506850 | Radish |
| SRR13213273 | PRJNA506850 | Radish |
| SRR13213276 | PRJNA506850 | Radish |
| SRR13213283 | PRJNA506850 | Radish |
| SRR13213288 | PRJNA506850 | Radish |
| SRR13213272 | PRJNA506850 | Radish |
| SRR13213279 | PRJNA506850 | Radish |
| SRR10585541 | PRJNA593573 | Brassica_oleracea |
| SRR10585559 | PRJNA593573 | Brassica_rapa |
| SRR10585542 | PRJNA593573 | Brassica_oleracea |
| SRR10585543 | PRJNA593573 | Brassica_oleracea |
| SRR10585544 | PRJNA593573 | Brassica_oleracea |
| SRR10585518 | PRJNA593573 | Amaranthus |
| SRR10585507 | PRJNA593573 | Amaranthus |
| SRR10585560 | PRJNA593573 | Brassica_rapa |
| SRR10585561 | PRJNA593573 | Brassica_rapa |
| SRR10585529 | PRJNA593573 | Amaranthus |
| SRR10585558 | PRJNA593573 | Brassica_rapa |
| SRR10585499 | PRJNA593573 | Brassica_oleracea |
| SRR10585540 | PRJNA593573 | Amaranthus |
| SRR10585498 | PRJNA593573 | Brassica_oleracea |
| SRR10585495 | PRJNA593573 | Brassica_oleracea |
| SRR10585497 | PRJNA593573 | Brassica_oleracea |
| SRR10585514 | PRJNA593573 | Brassica_rapa |
| SRR10585516 | PRJNA593573 | Brassica_rapa |
| SRR10585515 | PRJNA593573 | Brassica_rapa |
| SRR10585527 | PRJNA593573 | Amaranthus |
| SRR10585528 | PRJNA593573 | Amaranthus |
| SRR10585530 | PRJNA593573 | Amaranthus |
| SRR10585531 | PRJNA593573 | Amaranthus |
| SRR10585513 | PRJNA593573 | Brassica_rapa |
| A466 | PRJNA734564 | Apple |
| A465 | PRJNA734564 | Apple |
| A462 | PRJNA734564 | Apple |
| A464 | PRJNA734564 | Apple |
| A467 | PRJNA734564 | Apple |
| A463 | PRJNA734564 | Apple |

Supplementary Table S4.

List of accession numbers of gut metagenome samples from 12 studies that used to initially evaluate the presence of fruit and vegetable associated bacteria in the human gut

| SRA | Accession study | Reference |
| --- | --- | --- |
| ERR3277230 | PRJEB32135 | Cait, A., Cardenas, E., Dimitriu, P. A., Amenyogbe, N., Dai, D., Cait, J., ... & Mohn, W. W. (2019). Reduced genetic potential for butyrate fermentation in the gut microbiome of infants who develop allergic sensitization. Journal of Allergy and Clinical Immunology, 144(6), 1638-1647. |
| ERR3277229 | PRJEB32135 |  |
| ERR3277233 | PRJEB32135 |  |
| ERR3277235 | PRJEB32135 |  |
| ERR3277282 | PRJEB32135 |  |
| ERR3277261 | PRJEB32135 |  |
| ERR3277237 | PRJEB32135 |  |
| ERR3277303 | PRJEB32135 |  |
| ERR3277266 | PRJEB32135 |  |
| ERR3277300 | PRJEB32135 |  |
| ERR3277223 | PRJEB32135 |  |
| ERR3277224 | PRJEB32135 |  |
| ERR3277299 | PRJEB32135 |  |
| ERR3277275 | PRJEB32135 |  |
| ERR3277218 | PRJEB32135 |  |
| ERR3277227 | PRJEB32135 |  |
| ERR3277371 | PRJEB32135 |  |
| ERR3277226 | PRJEB32135 |  |
| ERR866570 | PRJEB9150 | David, L. A., Weil, A., Ryan, E. T., Calderwood, S. B., Harris, J. B., Chowdhury, F., ... & Turnbaugh, P. J. (2015). Gut microbial succession follows acute secretory diarrhea in humans. *MBio*, *6*(3), e00381-15. |
| ERR866567 | PRJEB9150 |  |
| ERR866561 | PRJEB9150 |  |
| ERR866563 | PRJEB9150 |  |
| ERR866564 | PRJEB9150 |  |
| ERR866568 | PRJEB9150 |  |
| ERR866569 | PRJEB9150 |  |
| ERR866565 | PRJEB9150 |  |
| ERR866562 | PRJEB9150 |  |
| ERR866571 | PRJEB9150 |  |
| ERR866566 | PRJEB9150 |  |
| ERR866572 | PRJEB9150 |  |
| ERR688561 | PRJEB7774 | Feng, Q., Liang, S., Jia, H., Stadlmayr, A., Tang, L., Lan, Z., ... & Wang, J. (2015). Gut microbiome development along the colorectal adenoma–carcinoma sequence. Nature communications, 6(1), 6528. |
| ERR688554 | PRJEB7774 |  |
| ERR688505 | PRJEB7774 |  |
| ERR688510 | PRJEB7774 |  |
| ERR688521 | PRJEB7774 |  |
| ERR688541 | PRJEB7774 |  |
| ERR688552 | PRJEB7774 |  |
| ERR688563 | PRJEB7774 |  |
| ERR688534 | PRJEB7774 |  |
| ERR688548 | PRJEB7774 |  |
| ERR688507 | PRJEB7774 |  |
| ERR688533 | PRJEB7774 |  |
| ERR688535 | PRJEB7774 |  |
| ERR688551 | PRJEB7774 |  |
| ERR688516 | PRJEB7774 |  |
| ERR688567 | PRJEB7774 |  |
| ERR688529 | PRJEB7774 |  |
| ERR688547 | PRJEB7774 |  |
| ERR688553 | PRJEB7774 |  |
| ERR688558 | PRJEB7774 |  |
| ERR688573 | PRJEB7774 |  |
| ERR688577 | PRJEB7774 |  |
| ERR688517 | PRJEB7774 |  |
| ERR688525 | PRJEB7774 |  |
| ERR688569 | PRJEB7774 |  |
| ERR688574 | PRJEB7774 |  |
| ERR688528 | PRJEB7774 |  |
| ERR688559 | PRJEB7774 |  |
| ERR688565 | PRJEB7774 |  |
| ERR2855797 | PRJEB29127 | Zhu, F., Ju, Y., Wang, W., Wang, Q., Guo, R., Ma, Q., ... & Ma, X. (2020). Metagenome-wide association of gut microbiome features for schizophrenia. *Nature communications*, *11*(1), 1612. |
| ERR2855810 | PRJEB29127 |  |
| ERR2855833 | PRJEB29127 |  |
| ERR2855816 | PRJEB29127 |  |
| ERR2855812 | PRJEB29127 |  |
| ERR2855789 | PRJEB29127 |  |
| ERR2855799 | PRJEB29127 |  |
| ERR2855807 | PRJEB29127 |  |
| ERR2855795 | PRJEB29127 |  |
| ERR2855831 | PRJEB29127 |  |
| ERR2855834 | PRJEB29127 |  |
| ERR2855846 | PRJEB29127 |  |
| ERR2855793 | PRJEB29127 |  |
| ERR2855820 | PRJEB29127 |  |
| ERR2855821 | PRJEB29127 |  |
| ERR2855811 | PRJEB29127 |  |
| ERR2855792 | PRJEB29127 |  |
| ERR2855794 | PRJEB29127 |  |
| ERR2855840 | PRJEB29127 |  |
| ERR2855842 | PRJEB29127 |  |
| ERR2855798 | PRJEB29127 |  |
| ERR2855801 | PRJEB29127 |  |
| ERR2855818 | PRJEB29127 |  |
| ERR2855800 | PRJEB29127 |  |
| ERR2855822 | PRJEB29127 |  |
| ERR2855786 | PRJEB29127 |  |
| ERR2855790 | PRJEB29127 |  |
| ERR2855802 | PRJEB29127 |  |
| ERR2855804 | PRJEB29127 |  |
| ERR2855806 | PRJEB29127 |  |
| ERR2855808 | PRJEB29127 |  |
| ERR2855815 | PRJEB29127 |  |
| ERR2855788 | PRJEB29127 |  |
| ERR2855814 | PRJEB29127 |  |
| ERR2855823 | PRJEB29127 |  |
| ERR2855809 | PRJEB29127 |  |
| ERR2855813 | PRJEB29127 |  |
| ERR2855819 | PRJEB29127 |  |
| ERR2855803 | PRJEB29127 |  |
| ERR2855805 | PRJEB29127 |  |
| ERR2855824 | PRJEB29127 |  |
| ERR2855817 | PRJEB29127 |  |
| SRR3313064 | PRJNA289586 | Heintz-Buschart, A., May, P., Laczny, C. C., Lebrun, L. A., Bellora, C., Krishna, A., ... & Wilmes, P. (2016). Integrated multi-omics of the human gut microbiome in a case study of familial type 1 diabetes. Nature microbiology, 2(1), 1-13. |
| SRR3313075 | PRJNA289586 |  |
| SRR3313066 | PRJNA289586 |  |
| SRR3313043 | PRJNA289586 |  |
| SRR3313078 | PRJNA289586 |  |
| SRR3313059 | PRJNA289586 |  |
| SRR3313060 | PRJNA289586 |  |
| SRR3313058 | PRJNA289586 |  |
| SRR3313077 | PRJNA289586 |  |
| SRR3313113 | PRJNA289586 |  |
| SRR3313061 | PRJNA289586 |  |
| SRR3313090 | PRJNA289586 |  |
| SRR8114103 | PRJNA289586 |  |
| SRR3313051 | PRJNA289586 |  |
| SRR3313069 | PRJNA289586 |  |
| SRR3313079 | PRJNA289586 |  |
| SRR8114116 | PRJNA289586 |  |
| SRR3313055 | PRJNA289586 |  |
| SRR3313068 | PRJNA289586 |  |
| SRR3313102 | PRJNA289586 |  |
| SRR3313040 | PRJNA289586 |  |
| SRR3313052 | PRJNA289586 |  |
| SRR3313056 | PRJNA289586 |  |
| SRR3313034 | PRJNA289586 |  |
| SRR3313057 | PRJNA289586 |  |
| SRR8114137 | PRJNA289586 |  |
| SRR8114100 | PRJNA289586 |  |
| SRR3313123 | PRJNA289586 |  |
| SRR8114106 | PRJNA289586 |  |
| SRR8090989 | PRJNA497734 | Kostic, A. D., Gevers, D., Siljander, H., Vatanen, T., Hyötyläinen, T., Hämäläinen, A. M., ... & DIABIMMUNE Study Group. (2015). The dynamics of the human infant gut microbiome in development and in progression toward type 1 diabetes. Cell host & microbe, 17(2), 260-273. |
| SRR8090638 | PRJNA497734 |  |
| SRR8085972 | PRJNA497734 |  |
| SRR8085971 | PRJNA497734 |  |
| SRR8086470 | PRJNA497734 |  |
| SRR8085970 | PRJNA497734 |  |
| SRR8086349 | PRJNA497734 |  |
| SRR8086421 | PRJNA497734 |  |
| SRR8090735 | PRJNA497734 |  |
| SRR8086308 | PRJNA497734 |  |
| SRR8086383 | PRJNA497734 |  |
| SRR8086463 | PRJNA497734 |  |
| SRR8086106 | PRJNA497734 |  |
| SRR8086108 | PRJNA497734 |  |
| SRR8086172 | PRJNA497734 |  |
| SRR8086309 | PRJNA497734 |  |
| SRR8086472 | PRJNA497734 |  |
| SRR8086473 | PRJNA497734 |  |
| SRR8086499 | PRJNA497734 |  |
| SRR8086033 | PRJNA497734 |  |
| SRR8086537 | PRJNA497734 |  |
| SRR8086218 | PRJNA497734 |  |
| SRR8086284 | PRJNA497734 |  |
| SRR8086468 | PRJNA497734 |  |
| SRR8086501 | PRJNA497734 |  |
| SRR8085973 | PRJNA497734 |  |
| SRR8086465 | PRJNA497734 |  |
| SRR8086471 | PRJNA497734 |  |
| SRR8090524 | PRJNA497734 |  |
| SRR8086469 | PRJNA497734 |  |
| SRR8090525 | PRJNA497734 |  |
| SRR8090742 | PRJNA497734 |  |
| SRR8090814 | PRJNA497734 |  |
| SRR8086467 | PRJNA497734 |  |
| SRR8090740 | PRJNA497734 |  |
| SRR8086123 | PRJNA497734 |  |
| SRR8090734 | PRJNA497734 |  |
| SRR8086502 | PRJNA497734 |  |
| SRR8090743 | PRJNA497734 |  |
| SRR8086464 | PRJNA497734 |  |
| SRR8086466 | PRJNA497734 |  |
| SRR8086035 | PRJNA497734 |  |
| SRR8086034 | PRJNA497734 |  |
| SRR8086032 | PRJNA497734 |  |
| SRR8086244 | PRJNA497734 |  |
| SRR8086406 | PRJNA497734 |  |
| ERR2506003 | PRJEB24006 | Levan, S. R., Stamnes, K. A., Lin, D. L., Panzer, A. R., Fukui, E., McCauley, K., ... & Lynch, S. V. (2019). Elevated faecal 12, 13-diHOME concentration in neonates at high risk for asthma is produced by gut bacteria and impedes immune tolerance. Nature microbiology, 4(11), 1851-1861. |
| ERR2506005 | PRJEB24006 |  |
| ERR2506004 | PRJEB24006 |  |
| ERR2506000 | PRJEB24006 |  |
| ERR2505988 | PRJEB24006 |  |
| ERR2505983 | PRJEB24006 |  |
| ERR2505997 | PRJEB24006 |  |
| ERR2505982 | PRJEB24006 |  |
| ERR2505986 | PRJEB24006 |  |
| ERR2505987 | PRJEB24006 |  |
| ERR2505994 | PRJEB24006 |  |
| ERR2505984 | PRJEB24006 |  |
| ERR2505990 | PRJEB24006 |  |
| ERR2505999 | PRJEB24006 |  |
| ERR2505995 | PRJEB24006 |  |
| ERR2505980 | PRJEB24006 |  |
| ERR2505985 | PRJEB24006 |  |
| ERR2505981 | PRJEB24006 |  |
| ERR2506001 | PRJEB24006 |  |
| ERR2505989 | PRJEB24006 |  |
| ERR2505992 | PRJEB24006 |  |
| ERR2505993 | PRJEB24006 |  |
| SRR2145331 | SRP057027 | Lewis, J. D., Chen, E. Z., Baldassano, R. N., Otley, A. R., Griffiths, A. M., Lee, D., ... & Bushman, F. D. (2015). Inflammation, antibiotics, and diet as environmental stressors of the gut microbiome in pediatric Crohn’s disease. *Cell host & microbe*, *18*(4), 489-500. |
| SRR2145355 | SRP057027 |  |
| SRR2145404 | SRP057027 |  |
| SRR2145291 | SRP057027 |  |
| SRR2145335 | SRP057027 |  |
| SRR2145351 | SRP057027 |  |
| SRR2145370 | SRP057027 |  |
| SRR2145372 | SRP057027 |  |
| SRR2145367 | SRP057027 |  |
| SRR2145368 | SRP057027 |  |
| SRR2145359 | SRP057027 |  |
| SRR2145369 | SRP057027 |  |
| SRR2145392 | SRP057027 |  |
| SRR2145347 | SRP057027 |  |
| SRR2145362 | SRP057027 |  |
| SRR2145365 | SRP057027 |  |
| SRR2145307 | SRP057027 |  |
| SRR2145339 | SRP057027 |  |
| SRR2145371 | SRP057027 |  |
| SRR2145363 | SRP057027 |  |
| SRR2145295 | SRP057027 |  |
| SRR2145388 | SRP057027 |  |
| SRR2145343 | SRP057027 |  |
| SRR2145361 | SRP057027 |  |
| SRR2145299 | SRP057027 |  |
| SRR2145360 | SRP057027 |  |
| SRR2145366 | SRP057027 |  |
| SRR2145364 | SRP057027 |  |
| SRR2145373 | SRP057027 |  |
| ERR209700 | PRJEB1220 | Nielsen, H. B., Almeida, M., Juncker, A. S., Rasmussen, S., Li, J., Sunagawa, S., ... & Ehrlich, S. D. (2014). Identification and assembly of genomes and genetic elements in complex metagenomic samples without using reference genomes. Nature biotechnology, 32(8), 822-828. |
| ERR209332 | PRJEB1220 |  |
| ERR209710 | PRJEB1220 |  |
| ERR209327 | PRJEB1220 |  |
| ERR209708 | PRJEB1220 |  |
| ERR209475 | PRJEB1220 |  |
| ERR209696 | PRJEB1220 |  |
| ERR209698 | PRJEB1220 |  |
| ERR209729 | PRJEB1220 |  |
| ERR209262 | PRJEB1220 |  |
| ERR209730 | PRJEB1220 |  |
| ERR209701 | PRJEB1220 |  |
| ERR209711 | PRJEB1220 |  |
| ERR209339 | PRJEB1220 |  |
| ERR209514 | PRJEB1220 |  |
| ERR209709 | PRJEB1220 |  |
| ERR209715 | PRJEB1220 |  |
| ERR209250 | PRJEB1220 |  |
| ERR209255 | PRJEB1220 |  |
| ERR209515 | PRJEB1220 |  |
| ERR209697 | PRJEB1220 |  |
| ERR209699 | PRJEB1220 |  |
| ERR209716 | PRJEB1220 |  |
| ERR209717 | PRJEB1220 |  |
| ERR209474 | PRJEB1220 |  |
| ERR209695 | PRJEB1220 |  |
| ERR209692 | PRJEB1220 |  |
| ERR209693 | PRJEB1220 |  |
| ERR209465 | PRJEB1220 |  |
| ERR209469 | PRJEB1220 |  |
| ERR209477 | PRJEB1220 |  |
| ERR209706 | PRJEB1220 |  |
| ERR209707 | PRJEB1220 |  |
| ERR209470 | PRJEB1220 |  |
| ERR209476 | PRJEB1220 |  |
| ERR209478 | PRJEB1220 |  |
| ERR209473 | PRJEB1220 |  |
| ERR209254 | PRJEB1220 |  |
| ERR209472 | PRJEB1220 |  |
| ERR209517 | PRJEB1220 |  |
| SRR9292917 | PRJNA547591 | Rubel, M. A., Abbas, A., Taylor, L. J., Connell, A., Tanes, C., Bittinger, K., ... & Tishkoff, S. A. (2020). Lifestyle and the presence of helminths is associated with gut microbiome composition in Cameroonians. Genome biology, 21, 1-32. |
| SRR9292753 | PRJNA547591 |  |
| SRR9292880 | PRJNA547591 |  |
| SRR9293015 | PRJNA547591 |  |
| SRR9293125 | PRJNA547591 |  |
| SRR9292739 | PRJNA547591 |  |
| SRR9292841 | PRJNA547591 |  |
| SRR9292909 | PRJNA547591 |  |
| SRR9292966 | PRJNA547591 |  |
| SRR9293131 | PRJNA547591 |  |
| SRR9293405 | PRJNA547591 |  |
| SRR9293373 | PRJNA547591 |  |
| SRR9293399 | PRJNA547591 |  |
| SRR9292883 | PRJNA547591 |  |
| SRR9293337 | PRJNA547591 |  |
| SRR9292679 | PRJNA547591 |  |
| SRR9293458 | PRJNA547591 |  |
| SRR5763462 | PRJNA392180 | Smits, S. A., Leach, J., Sonnenburg, E. D., Gonzalez, C. G., Lichtman, J. S., Reid, G., ... & Sonnenburg, J. L. (2017). Seasonal cycling in the gut microbiome of the Hadza hunter-gatherers of Tanzania. Science, 357(6353), 802-806. |
| SRR5763458 | PRJNA392180 |  |
| SRR5763461 | PRJNA392180 |  |
| SRR5763456 | PRJNA392180 |  |
| SRR5763459 | PRJNA392180 |  |
| SRR5763460 | PRJNA392180 |  |
| SRR5763448 | PRJNA392180 |  |
| SRR5763457 | PRJNA392180 |  |
| SRR5763445 | PRJNA392180 |  |
| SRR5763447 | PRJNA392180 |  |
| SRR5763455 | PRJNA392180 |  |
| SRR5763446 | PRJNA392180 |  |
| SRR5763449 | PRJNA392180 |  |
| SRR5763450 | PRJNA392180 |  |
| SRR5763452 | PRJNA392180 |  |
| SRR5763451 | PRJNA392180 |  |
| SRR5763453 | PRJNA392180 |  |
| SRR5763454 | PRJNA392180 |  |
| ERR911962 | PRJEB9576 | Xie, H., Guo, R., Zhong, H., Feng, Q., Lan, Z., Qin, B., ... & Jia, H. (2016). Shotgun metagenomics of 250 adult twins reveals genetic and environmental impacts on the gut microbiome. Cell systems, 3(6), 572-584. |
| ERR912068 | PRJEB9576 |  |
| ERR912038 | PRJEB9576 |  |
| ERR912103 | PRJEB9576 |  |
| ERR912202 | PRJEB9576 |  |
| ERR911965 | PRJEB9576 |  |
| ERR912045 | PRJEB9576 |  |
| ERR912190 | PRJEB9576 |  |
| ERR912126 | PRJEB9576 |  |
| ERR912134 | PRJEB9576 |  |
| ERR912181 | PRJEB9576 |  |
| ERR911979 | PRJEB9576 |  |
| ERR912004 | PRJEB9576 |  |
| ERR912092 | PRJEB9576 |  |
| ERR912124 | PRJEB9576 |  |
| ERR911992 | PRJEB9576 |  |
| ERR912041 | PRJEB9576 |  |
| ERR912043 | PRJEB9576 |  |
| ERR912100 | PRJEB9576 |  |
| ERR912112 | PRJEB9576 |  |
| ERR912136 | PRJEB9576 |  |
| ERR912146 | PRJEB9576 |  |
| ERR912201 | PRJEB9576 |  |
| ERR911984 | PRJEB9576 |  |
| ERR911996 | PRJEB9576 |  |
| ERR912084 | PRJEB9576 |  |
| ERR912085 | PRJEB9576 |  |
| ERR912113 | PRJEB9576 |  |
| ERR912122 | PRJEB9576 |  |
| ERR911955 | PRJEB9576 |  |
| ERR911976 | PRJEB9576 |  |
| ERR912029 | PRJEB9576 |  |
| ERR912077 | PRJEB9576 |  |
| ERR912089 | PRJEB9576 |  |
| ERR912148 | PRJEB9576 |  |
| ERR912180 | PRJEB9576 |  |
| ERR912189 | PRJEB9576 |  |
| ERR911990 | PRJEB9576 |  |
| ERR911997 | PRJEB9576 |  |
| ERR912030 | PRJEB9576 |  |
| ERR912033 | PRJEB9576 |  |
| ERR912046 | PRJEB9576 |  |
| ERR912075 | PRJEB9576 |  |
| ERR912101 | PRJEB9576 |  |
| ERR912133 | PRJEB9576 |  |
| ERR912178 | PRJEB9576 |  |
| ERR912020 | PRJEB9576 |  |
| ERR912086 | PRJEB9576 |  |
| ERR912097 | PRJEB9576 |  |
| ERR912151 | PRJEB9576 |  |
| ERR912166 | PRJEB9576 |  |
| ERR911993 | PRJEB9576 |  |

Supplementary Data S1. (separate file)

Presence of putative health-promoting genes in bacterial metagenome assembled genomes that were recovered from fruits and vegetables
